# Rhizoplane and Rhizosphere Fungal Communities of Geographically Isolated Korean Bellflower (*Campanula takesimana* Nakai)

**DOI:** 10.3390/biology10020138

**Published:** 2021-02-10

**Authors:** Jong Myong Park, Bomi Kim, Young-Chang Cho, Byoung-Hee Lee, Ji Won Hong, Young-Hyun You

**Affiliations:** 1 Quality Research Institute, Waterworks Headquarters Incheon Metropolitan City, Incheon 22101, Korea; eveningwater@hanmail.net; 2 Microorganism Resources Division, National Institute of Biological Resources, Incheon 22689, Korea; htes0311@korea.kr; 3 College of Pharmacy, Chonnam National University, Gwangju 61186, Korea; yccho@chonnam.ac.kr; 4 Biological and Genetic Resources Assessment Division, National Institute of Biological Resources, Incheon 22689, Korea; dpt510@korea.kr; 5 Department of Hydrogen and Renewable Energy, Kyungpook National University, Daegu 41566, Korea

**Keywords:** Korean bellflower, *Campanula takesimana*, geographical segregation, *Mortierella*, rhizoplane, rhizosphere

## Abstract

**Simple Summary:**

The current study reports fungal diversities in the rhizoplane (RP) and rhizosphere (RS) samples of the geographically isolated Korean bellflower (*Campanula* *takesimana*) obtained from its original habitats of the eastern coast of the Korean Peninsula for the first time. The identification of specific taxa in each site may provide a better understanding of the interaction between the soil fungi and Korean bellflower.

**Abstract:**

Fungal communities in the rhizoplane (RP) and rhizosphere (RS) of geographically isolated *C. takesimana* habitats in different environments such as oceanic (Seodo, the Dokdo Islands), coastline (Sadong, Ulleungdo Island), and inland (Taeha, Ulleungdo Island) regions were analyzed by MiSeq sequencing. In total, 1279 operational taxonomic units (OTUs) were obtained and they were further classified into 185 genera belonging to five phyla. The total number of fungal taxa in the RP samples was lower than those in the RS samples in all the sampled locations, providing an indication of the existence of a certain level of the selective pressures from the host plant. The richness of the RP in the Dokdo Islands was higher than that of Ulleungdo Island, but the richness of the RS in the Dokdo Islands was lower than that of Ulleungdo Island. These results suggest evidence for strong effects of a harsh geo-climate on the RP and RS fungal diversities in the Dokdo Islands. Additionally, a total of 82 fungal genera were identified in all three RP samples and 63 genera (77%) were uniquely found in each of the geographical regions and 43 genera (52.4%) showed high dependency on the *C. takesimana* vegetation. It was found that the genus *Mortierella* was the most dominant taxon in all the samples. The geo-ecological isolation of the Korean bellflower may have caused unique formation of the RP and RS fungal communities in the natural habitats.

## 1. Introduction

There are 84 herbaceous plant species belonging to the family Campanulaceae (bellflower family) that are native to the East Asia and the Korean Peninsula [1]. Many of them, such as *Platycodon grandiflorus* (Jacq.) A.DC., *Codonopsis lanceolata* (Siebold and Zucc.) Benth. and Hook.f. ex Trautv., *Campanula punctata* Lam., and *Campanula takesimana*, are reported to have a variety of pharmacological effects on respiratory or metabolic diseases [2,3,4,5,6]. *Campanula punctata* is native to the inland terrain of the Korean Peninsula, while *C. takesimana*, commonly known as the Korean bellflower, is endemic only to Ulleung Island, located in the East Sea of Korea [1]. Recently, a new *C. takesimana* community was also found on Seodo (the West Island), one of the Dokdo Islands, which has served as home to many wild halotolerant plants [7,8].

*Campanula takesimana* shows slight morphological differences compared to *C. punctata*. Its distinctive light pink or purple petals are the key taxonomic feature of *C. takesimana* which has selectively survived and evolved only on the volcanic islands of Korea [1,9,10]. Based on the internal databases of the Korean National Institute of Biological Resources (NIBR), the Korean bellflower is defined as a halotolerant, herbaceous, and perennial species that only grows naturally in the maritime terrains of the Korean Peninsula [7]. Its colonies are usually found on cliffs and sloping ground adjacent to the sea and a fully grown colony is 30–100 cm tall [11].

Historically, stems, leaves, and flowers of *C. takesimana*, except for roots, have long been used in Korea as food ingredients and food additives. Furthermore, pharmacologically active compounds such as chlorogenic acid have also been identified in *C. takesimana*. It is presumed that its unique antioxidant traits have resulted from a gradual adaptation to the harsh marine environment of these oceanic islands since mycorrhizal symbiosis may alter phenolic acid content in plants and the host plants may establish an efficient way to increase stress tolerance under certain conditions through this mechanism [12,13,14].

It is well documented that geographical isolation causes species differentiation in higher life forms and the bellflower species has been extensively studied as a model organism for geological segregation research since it has a high adaptability to divergent environment [12,15]. It has also been reported that the adaptation and flourishing of higher plants in a barren maritime terrain are, to some extent, driven by the symbiotic relationships between soil fungi and plants [16]. However, there have been no clear answers to how fungal diversity differ between geographically isolated host bellflower species and how fungal communities shift in the course of adaption to given environmental circumstances. Thus, more intensive research on microbial communities is needed to define and eventually secure symbiotic microbial resources of *C. takesimana* in these remote oceanic islands of Korea [8].

Hence, rhizoplane (RP; where ectomycorrhizas strongly interact with the host plants) and rhizosphere (RS; soil away from areas directly affected by plant roots) fungal communities from the three different geographically isolated natural habitats of the Korean bellflowers were analyzed by culture-independent methods in order to identify fungal taxa associated with *C. takesimana* and to reveal how geographical segregation of the host affects the compositions of the RS and RP fungal communities. Therefore, the current research focuses on soil fungi analysis around the host plants with use of the recent next-generation sequencing technology so that the largest number of microbes present in the soil could be identified. It should be noted that pyrosequencing may not be a sufficient method to identify all inhabiting fungi since the technique may omit spores, fungi present in low amounts, or some fungi with different internal transcribed spacer (ITS) sequence regions [17].

## 2. Materials and Methods

**Figure 1:**
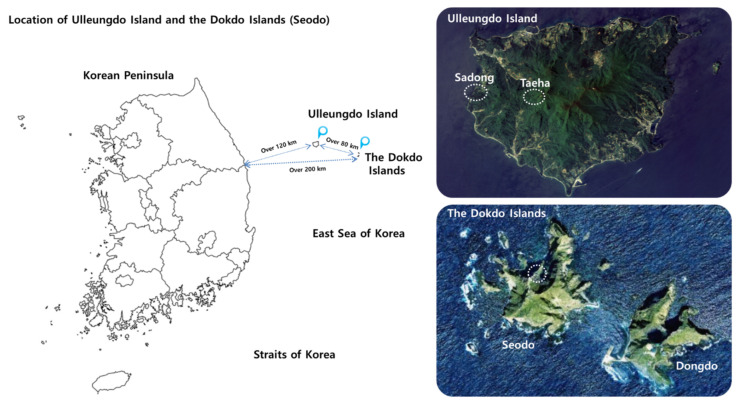
Geographically segregated natural habitats of the Korean bellflowers. The map images were downloaded from the Cultural Heritage Administration of Korea (CHA) Portal Site (http://www.heritage.go.kr/heri/idx/index.do, accessed on 21 October 2020) and modified for this study.

The Korean bellflower is capable of surviving under both the extremely harsh conditions of volcanic ocean islands and relatively mild conditions of inland terrains of Korea. Therefore, to identify their natural RP and RS fungal components, the target sampling sites where no human activities are allowed or they are tightly controlled were carefully selected in order to correctly represent such environments. However, it is very difficult to find such places in the mainland of the Korean Peninsula. Hence, a virgin terrain on Ulleung Island that represents the inland geography with very limited human access was selected. Accordingly, the RP and RS samples of the Korean bellflower were collected from 3 geographically segregated sites: (1) the Dokdo Islands (Seodo), a rocky oceanic island with volcanic origin [7,18,19]; (2) the coastline of Ulleungdo Island, which has less harsh conditions than those of Seodo [19]; (3) the central inland area of Ulleungdo Island, which is surrounded by mountain ranges composed of virgin primeval forests (Figure 1 and Table 1) [19,20]. All these areas have been designated as National Reserve by the Cultural Heritage Administration of Korea (CHA) for the purpose of conservation [7]. These sampling sites are geographically isolated and segregated from the mainland of Korea and the Japanese archipelago, respectively [7]. It is generally assumed that no ecological or genetic exchanges by higher animals that can travel long distances have occurred. The primary habitats of the Korean bellflower are cliffs and slopes [11] where human access is almost impossible. Although the origin of the Korean bellflower still remains enigmatic, it is generally believed that *C. takesimana* is a native species to the Dokdo Islands and Ulleungdo Island.

### 2.1. Sampling of the Host Plant

The RP is the surface of the plant roots where soil microorganisms establish a mutualistic association with the host plant [21], while the RS represents the area of soil around plant roots where highly active interactions among soil, host plant, and microbiome takes place [22]. From each site, about 15–30 fully grown adult plants (15 from Seodo, 30 from Sadong, and 30 from Taeha, respectively) were taken and the topsoil around the roots were sampled in the center of their vegetation for the RP and RS, respectively. The roots of *C. takesimana* were agitated in sterile distilled water 3 times and the remaining parts were gathered as the RP samples. The RS soil core was taken to the depth of 30 cm with a diameter of 2 mm from each sampling site. Since soil loss due to steep slopes is often observed in Seodo [7], *C. takesimana* habitats with no signs of soil loss or soil movement were carefully selected for sampling. All the samplings were carried out from 25 to 28 April 2017.

### 2.2. DNA Extraction and Polymerase Chain Reaction Amplification

All samples were transported to the laboratory at the NIBR for DNA extraction. The soil samples were pooled to obtain a 1 kg composite sample per site. Then, 0.5 g of each sample was used for DNA extraction with a PowerSoil DNA isolation kit (Mo Bio Laboratories, Carlsbad, CA, USA) according to the manufacturer’s protocol. The fungal ITS1 was amplified using the ITS1F (5′-CTT GGT CAT TTA GAG GAA GTA A-3′) and ITS2 (5′-GCT GCG TTC TTC ATC GAT GC-3′) pair of primers [23,24]. The resulting ITS1 amplicons were sequenced at Macrogen (Seoul, Korea) using the paired-end (2 × 300 nt) Illumina MiSeq sequencing system (Illumina, San Diego, CA, USA).

### 2.3. Sequence Processing and Data Analyses

The paired-end sequences were assembled using PANDAseq software [25]. After assembly, all the sequence data were processed using the Mothur pipeline [26]. For fungal community analysis, the flanking gene fragments were removed from the ITS1 region using ITSx 1.0.9 software [27]. Putative chimeric sequences were detected and removed by the chimera.uchime algorithm available within Mothur [28] in de novo mode. The taxonomic classification was performed using Mothur’s version of the naïve Bayesian classifier, using the UNITE database for fungi [29]. QIIME implementation of UCLUST [30,31] was used to assign the operational taxonomic units (OTUs), defined with a limit threshold of 97% sequence similarity for fungi. All singleton OTUs were removed from the datasets prior to analysis. All samples were standardized by random subsampling using the sub.sample command (http://www.Mothur.org/wiki/Sub.sample, accessed on 7 March 2020) in Mothur. Chao’s richness, Shannon index, Simpson’s diversity index, and rarefaction values [32] were also estimated by using QIIME [33,34].

## 3. Results and Discussion

### 3.1. Illumina MiSeq Sequencing Results

Firstly, the OTU values and fungal genera identified in the RS samples were higher than those of the RP samples in all the geographical regions (Table 2). Secondly, the RP/RS ratio values of the OTU and genus in the coastline and inland regions of Ulleungdo Island were lower than those of the Dokdo Islands. Lastly, the numbers of the OTUs and fungal genera detected in the RP of *C. takesimana* from the Dokdo Islands were the highest among all the RP samples.

*C. takesimana* is resistant to high salt content that constantly flows from the marine environment. However, it is not capable of regulating sodium ions entering their xylem stream unlike other halophytes [35]. On the other hand, the Korean bellflower has certain physiological advantages since they can switch from C_3_ to C_4_ or crassulacean acid metabolism photosynthesis under unfavorable conditions such as drought stress [36,37]. Additionally, *Campanula* species, including *C. takesimana*, are articulated laticifers which produce multiple secretions and large amounts of organic matter into their root layers. This property may promote mycorrhizal establishment or functionality of salt-resistant heterotrophic fungal species in the RP and/or RS of *C. takesimana*.

### 3.2. Variation of the Fungal Phyla Associated with Geographically Isolated Hosts

**Figure 2:**
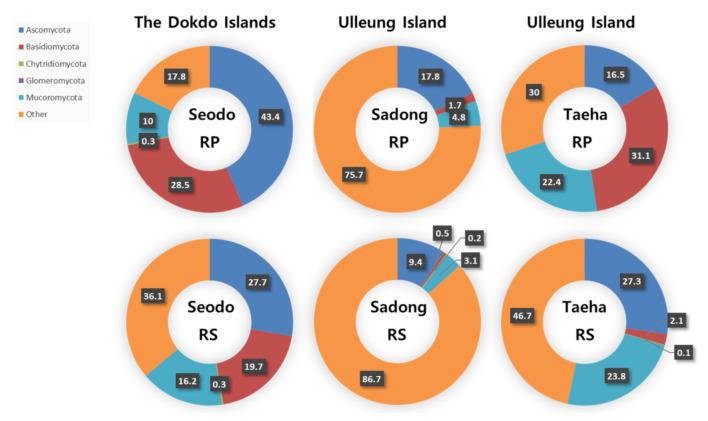
Fungal phyla distribution in the rhizoplane (RP) and rhizosphere (RS) samples (unit: %).

As shown in Figure 2, the most dominant fungal phylum across all the Dokdo Islands samples was Ascomycota (RP: 43.4% and RS: 27.7%). Ascomycota was also the most dominant phylum in Sadong, the coastline habitat of *C. takesimana* of Ulleungdo Island (RP: 17.8% and RS: 9.4%). Although there was a tendency of decline in the dominance of Ascomycota with the RS samples, it still remained the most dominant taxon in the Dokdo Islands and Sadong samples. On the other hand, the most abundant phylum in Taeha, the inland area of Ulleungdo Island, was Basidiomycota in the RP (31.1%), while Ascomycota was the second most dominant phylum (16.5%). The second most dominant phylum in the Dokdo Islands was Basidiomycota, whereas the dominance was lowered in the RS to 19.7%. Mucoromycota was the second most dominant phylum in Sadong (RP: 4.8% and RS: 3.1%) and Taeha (RP: 22.4% and RS: 23.8%), respectively.

In summary, Ascomycota was predominantly found in most of these segregated regions. However, the fungal phylum distribution in the RS varied considerably between the three sites since the RS is more susceptible than the RP to environmental influences. Additionally, the dominance of Ascomycota decreased or increased as the distance from the RP increased. In conclusion, no clear patterns were observed in the fungal phyla distribution of the soil samples.

### 3.3. Variation of Fungal Diverstity

**Figure 3:**
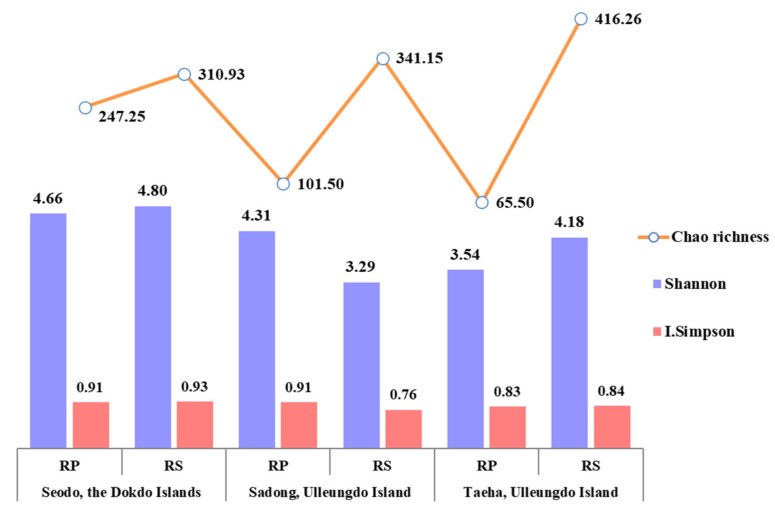
Fungal diversity variation in the RP and RS samples.

The diversity of fungi in the RP and RS of the host plant was analyzed using the Shannon index, Simpson’s diversity index, and Chao’s richness index. The Shannon index represents the evenness of species distribution and the Simpson’s index indicates the dominance of specific species in a particular environment, respectively [33]. Chao1 richness represents an estimate of the total number of species in the samples [34]. In this study, the RP and RS analysis results from each segregated region did not show any specific patterns associated with the geographical features (Figure 3). Additionally, there were no clear patterns in the dominance of certain fungal taxa in the samples. However, the species abundance showed the distinctive patterns with much higher values in the RS than in the RP in all three geographically segregated regions. The decrease in the fungal taxon abundance in the RP may suggest certain levels of selective pressures from the host [38]. Even though the clear species abundance patterns were shown in all the regions, the highest species abundance was observed in the natural habitat of *C. takesimana* on the Dokdo Islands. This could be interpreted as more diverse fungal communities being formed under more selective environmental pressure. It is also presumed that a higher fungal diversity in the RP compared to the RS might be the result of seed-associated endophytes, spread with the Korean bellflower seeds.

### 3.4. Variation of Fungal Genera

**Figure 4:**
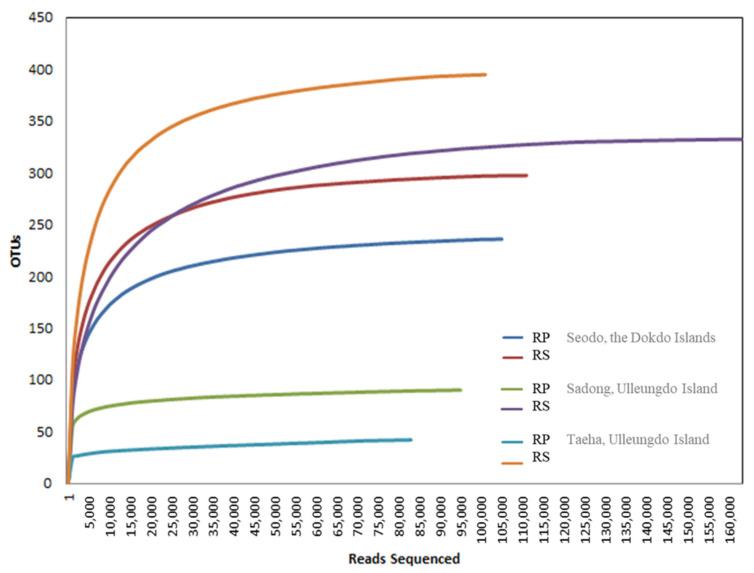
Illumina MiSeq sequencing rarefaction curve for operational taxonomic units (OTUs) from each site. OTUs were clustered at 3% dissimilarity using CD-HIT. The microbial community in the RP and the RS of Seodo, the Dokdo Islands (OTUs, 140/341; sequencing reads, 84,267/94,745), Sadong, coastline of Ulleungdo Island (OTUs, 154/389; sequencing reads, 95,395/117,939), and Taeha, inland of Ulleungdo Island (OTUs, 212/478; sequencing reads, 86,299/89,864), are shown.

**Figure 5:**
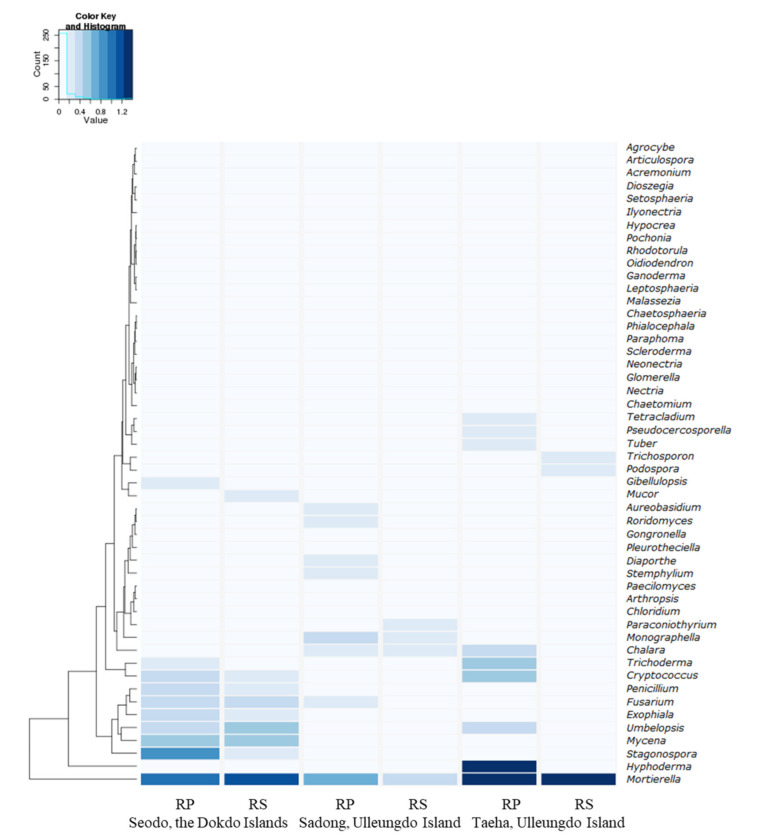
Heatmap of the 50 major fungal genera, including *Mortierella* species, distributed around host plants in each geographically segregated environment with a hierarchical clustering.

The main purpose of this study was to identify fungal diversity in the naturally occurring habitat of the Korean bellflower. To achieve this goal, Illumina MiSeq sequencing (Table 3 and Figure 4) and diversity data (Figure 3) analyses were used to determine the fungal taxon distribution of each geographically separated oceanic region (Figure 5). In total, 1279 OTUs at a 97% similarity level were obtained. In Seodo, the Dokdo Islands, 140/341 (RP/RS) OTUs were confirmed, in Sadong, coastline of Ulleungdo Island, 154/389 (RP/RS) OTUs were identified, and in Taeha, inland of Ulleungdo Island, 212/478 (RP/RS) OTUs were found, respectively (Figure 4).

The most dominant fungal genera in the RPs of the Dokdo Islands samples were *Mortierella* (7.78%), *Fusarium* (1.23%), and *Penicillium* (1.06%). The top three genera identified in the RPs of the Sadong samples were *Mortierella* (3.54%), *Fusarium* (0.54%), and *Trichoderma* (0.38%), while *Mortierella* (21.26%) and *Trichoderma* (2.21%) were predominant in the RPs of the Taeha samples (Table 3).

The genus *Mortierella* is known as a saprophyte that derives nutrients from decaying organic matters such as feces, trees, and insects [39,40]. In addition, *Penicillium* and *Trichoderma* are saprophytes [41], but they also play imperative roles in plant growth and nutrient absorption as well as resistance to extreme conditions, pathogens, and grazing by herbivores [42,43,44].

Regarding the difference between the RP and RS in the genus dominance, the most dominant genus, *Mortierella*, showed decreased percentages in the RPs of the Seodo and Taeha samples compared to the RSs, but the opposite result was observed in the Sadong samples. Therefore, there were no specific patterns in the dominance of *Mortierella* and other fungal genera including *Fusarium*, *Penicillium*, and *Trichoderma* (Table 3 and Figure 5).

### 3.5. Host Dependency and Selective Pressure

**Figure 6:**
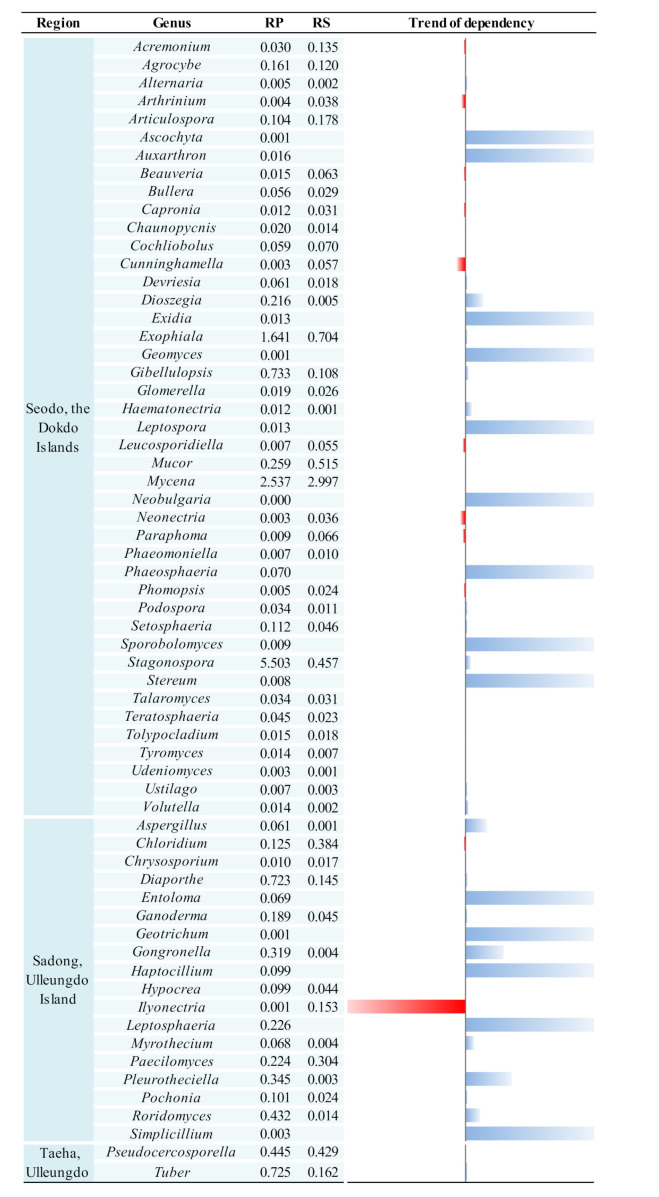
Unique fungal genera distributed in the RP and RS in each geographically isolated region and their host dependency. Blue bars indicate host dependency and red bars indicate increased dominance ratio as physical distance is away from the RP towards to the RS. Each number indicates the relative percentage of each genus and the trend of dependency of each genus was calculated by the following equation: (RS − RP)/RP.

The present study also aimed to compare and analyze the RP and RS fungal communities in the contrasting habitats of the Korean bellflower. It was confirmed that the *C. takesimana* RP exerted some selective pressures on their fungal clusters. Therefore, a comparative analysis of unique fungal taxon composition in the RP of *C. takesimana* from the three geographically separated regions may provide a better understanding of this phenomenon. A total of 82 fungal genera was identified in the three RP samples. There were 58 genera found in Seodo, 34 genera in Sadong, and 15 genera in Taeha, respectively. Among the 82 genera, 63 genera (77%) were uniquely found in each of the geographical region (43 genera in Seodo, 18 genera in Sadong, and 2 genera in Taeha, respectively) (Figure 6).

Meanwhile, the host dependency of each of the uniquely existing RP fungal genera could be identified by comparing the dominance variation between the RS and RP. If a particular taxon was detected only in the RP, but not in the RS, it was assumed that the taxon is dependent on the plant. Among the total unique genera, 43 genera (52.4%) showed a dependence on *C. takesimana* and it was presumed that a close interaction was established between these fungal taxa and the host plant. There were 27 RP-specific genera with high dependency in Seodo, 14 genera in Sadong, and 2 genera in Taeha, respectively. The fungal genera showing dependencies to the host plant’s RP are visualized in Figure 6. Especially, *Ascochyta*, *Auxarthron*, *Exidia*, *Geomyces*, *Leptospora*, *Neobulgaria*, *Phaeosphaeria*, *Sporobolomyces*, and *Stereum* were found only in the RP of Seodo, but they were not observed in the RS of Seodo. In addition, *Entoloma*, *Geotrichum*, *Haptocillium*, *Leptosphaeria*, and *Simplicillium* were found only in the RP of Sadong, but they were not identified in the RS of Sadong. These genera exhibited strong dependencies on the host plant. Therefore, it could be concluded that the ecological isolation of the Korean bellflower may have caused the formation of the unique fungal taxon that have strong interactions with *C. takesimana*.

The distribution of RS fungi was analyzed and, overall, 172 RS fungal genera were identified—71 genera in Seodo, 86 genera in Taeha, and 101 genera in Sadong were observed, respectively. The uniquely found genera in each site (30 genera from Seodo, 31 genera from Sadong, and 44 genera from Taeha, respectively) comprised 61% of all the identified RS fungal genera. The RS fungal genera that were not identified in the RP (13 genera in Seodo, 27 genera in Sadong, and 41 genera in Taeha, respectively) appear to be independent of the host plant.

In particular, the genus *Ilyonectria* (Figure 6), which was reported as a plant pathogen or an opportunistic pathogen [45,46,47,48], was significantly suppressed in the RP and its dominance increased steeply in the RS of Sadong (Figure 6). It was also reported that this genus participates in a combination of endophytic and saprotrophic activities of plants [49]. However, no conclusion can be drawn whether it was inhibited by the presence of *C. takesimana*’s roots since this genus was only identified in Sadong on Ulleungdo Island in this study.

The comparative analysis results showed that the genus *Stemphylium* was identified only in the RPs of Seodo and Sadong. It has been reported that *Stemphylium* exhibits pathogenicity to the family Campanulaceae, including *Platycodon grandiflorus* [50], which is a widely used alternative medicine in various regions, especially in Asia. The members of this genus include *S. alfalfae*, *S. bolickii*, *S. cannabinum*, *S. globuliferum*, *S. lycopersici*, *S. sarciniforme*, *S. solani*, and *S. vesicarium* [51] and they are not considered as plant symbionts. *Hyphoderma* was found in the RPs of Sadong and Taeha on Ulleungdo Island, but this genus was not present in the RSs of the same sampling sites. No conclusion can be made from this result as *Hyphoderma* is a ubiquitous corticioid homobasidiomycetes genus that have been commonly reported worldwide. The fungal genera that existed only in the RP of Seodo include *Ascochyta*, *Auxarthron*, *Exidia*, *Geomyces*, *Leptospora*, *Neobulgaria*, and *Stereum*. Even though their dominance was below 0.3% in the RP (Table S1), it seems that these fungal genera within the range of the selective pressures from *C. takesimana* vegetation are more competitive in this environment than other fungi. Some of the fungal genera only found in the RP are generally known for their pathogenicity to a wide range of plants. Information on the interdependency trends between the host species and microbes was obtained by comparing fungal genera in the RP and RS in this study. However, such dependencies cannot always be interpreted as symbiotic relationships [52] because certain pathogens usually have strong host (species) dependencies according to traditional pathobiology or plant pathophysiology [53,54]. *Ascochyta* is a genus of ascomycete fungi that contains several species that are pathogenic to crop species [55], but the interactions between *Auxarthron* species and plants are still unknown. *Exidia* is a genus of endogenous fungi that produce antioxidants [56] and no pathogenicity has yet been reported for this taxon. *Geomyces* is a genus of filamentous fungi belonging to the Myxotrichaceae family. Members of this genus are widely distributed, especially in northern temperate regions [57], and they are also known as psychrotolerant fungi associated with Arctic permafrost soils [58]. While the genus *Neobulgaria* usually grows on fallen dead beech trees, the genus *Stereum* is found on all kinds of deadwood, hardwood, or dead leaves [59] and they are sometimes observed on living tree leaves. *Leptospora* is a genus of fungi within the class Dothideomycetes and it is known as a nonpathogenic fungus [60]. As mentioned earlier, soil fungal diversity in the RP are primarily determined by the selective pressure derived from host plant species. Hence, both higher richness and species diversity may play vital roles in enhancing the stability of the corresponding soil fungal communities.

## 4. Conclusions

In this study, four fungal genera including *Fusarium*, *Mortierella*, *Penicillium*, and *Trichoderma* were commonly present in all the geographically segregated regions and they were distributed both in the RS and RP samples (Table 3). The genus *Mortierella* dominated the fungal communities in both the RP and RS and it showed the highest dominance among all the genera identified in this research. Furthermore, its dominance increased as the distance increased from the RPs towards the RSs in Seodo and Taeha. However, the opposite result was observed in the Sadong samples. The cause of this decrease in the fungal composition in the inland region of Ulleungdo Island is not clear. It was assumed that a kind of competition with other fungal genera rather than interaction with the host plant may have caused this since the RS is more susceptible to a variety of biotic and abiotic stresses. The genus *Mortierella* is usually nonpathogenic for plants, animals, and humans [61]. *M. wolfii* is the only *Mortierella* species that is pathogenic to humans and other animals and it is commonly isolated from soil, rotten silage, and similar substrates, often causing bovine abortion, pneumonia, and systemic mycosis [62]. However, given the remote conditions of the major habitats of the Korean bellflower, the human activity-induced pathogenicity of *Mortierella* with the host species is neglectable. Several Basidiomycota fungal genera have been reported to help their host plant species by taking up soil minerals from land via forming mycorrhizae at the roots of vascular plants under unfavorable conditions. *Mortierella*, in turn, obtains materials for survival synthesized by its host plants through photosynthesis [63,64]. Several *Mortierella* species have shown that they are able to confer enhanced tolerance to rot disease in *Crocus* corms [65] and increased resistance to salt stress in halophytic plants [66], respectively. It has also been reported that the *Mortierella* species promote their host plants’ growth by producing phyto-hormones [61]. *Mortierella* also exists as an ectrophic mycorrhiza in the roots of the bellflower, having a positive effect on the growth of the host species. The comparative analysis results indicated that there was no specific pattern of the variation of dominance depending on the distance from the host plant’s RP, but our study was able to show the distribution of *Mortierella* as the most dominant fungal genus present in the RP and RS of *C. takesimana*. It would be interesting for further research to investigate the ecological roles of *Mortierella* and other dominant fungi with respect to the plant’s growth and function and its adaptation to the harsh environments by means of transcriptomic analysis.

To our knowledge, this is the first report to investigate the fungal diversities in RP and RS samples of *C. takesimana* obtained from its natural habitats. The current results obtained by the analysis of both host dependency and selective pressure may enhance our understanding of *C. takesimana*’s ability to withstand the harsh environments of the Dokdo Islands and Ulleung Island since the identification of specific taxa in each site can provide a brief overview of the presence of the dominant RP and RS fungi in *C. takesimana* and this information would help us to narrow down future research on how these fungi function with the host plant under these extreme conditions. Lastly, it was reported that microbial communities may vary across seasons more than in response to long-term climate changes [67]. Hence, it would also be interesting to investigate the fungal diversities in the RP and RS of the Korean bellflower on a regular basis at specific times of the year.

## Figures and Tables

**Table 1:** Sampling site information.

Site	Coordinates (Latitude, Longitude)	Avg. Altitude (m)	Angle of Slope (°)	Geographical Feature	Avg. Height (cm)	Avg. Density (Individual/m^2^)	Surrounding Vegetation	Avg. Area Occupied by a Colony (m^2^)
Seodo, the Dokdo Islands	37°14′31.2″ N, 131°51′53.0″ E	87	45	Small oceanic volcanic island	5	20	Herbaceous plants	1
Sadong, Ulleungdo Island	37°27′23.6″ N, 130°52′30.0″ E	10	20	Coastline of large volcanic island	25	40	Herbaceous plants	9
Taeha, Ulleungdo Island	37°29′40.8″ N, 130°49′39.4″ E	425	5	Inland area of large volcanic island	15	80	Herbaceous plants	6

**Table 2:** Sampling site information comparison of Illumina MiSeq sequencing results.

Sampling Site	Seodo, the Dokdo Islands		Sadong, Ulleungdo Island		Taeha, Ulleungdo Island	
Sampling Point	RP	RS	RP	RS	RP	RS
Total read counts	240,956	247,847	194,918	285,563	160,662	238,724
Total bases	87,920,927	92,067,817	76,300,872	105,684,291	63,478,745	88,850,527
Number of valid sequences	103,130	105,988	93,402	142,825	78,860	95,022
GC (%)	49.7	49.6	51.8	52.9	47.8	49.0
OTUs	236	298	90	333	42	395
Fungal phylum	5	4	3	5	3	5
Fungal genera	58	71	34	85	15	101

**Table 3:** Variation of the major fungal genera.

Genus	Seodo, the Dokdo Islands		Sadong, Ulleungdo Island		Taeha, Ulleungdo Island	
	RP (%)	RS (%)	RP (%)	RS (%)	RP (%)	RS (%)
*Fusarium*	1.225	1.154	0.541	0.096	0.001	0.049
*Mortierella*	7.780	13.064	3.541	1.732	21.260	23.204
*Penicillium*	1.063	1.020	0.246	0.267	0.001	0.363
*Trichoderma*	0.778	0.104	0.386	0.320	2.205	0.155

## Data Availability

All the raw sequences obtained from this study were deposited at the National Center for Biotechnology Information (NCBI) Sequence Read Archive (SAR) under the project accession number PRJNA616069.

## Supplementary Materials

The following are available online at https://www.mdpi.com/2079-7737/10/2/138/s1, Table S1: Fungal genus distribution, Table S2: Soil analysis results.
