# Supplementary material for: Rhizoplane and Rhizosphere Fungal Communities of Geographically Isolated Korean Bellflower (Campanula takesimana Nakai)
Source: Biology (Basel). 2021 Feb 10;10(2):138. doi: 10.3390/biology10020138 (PMC7916508; doi:10.3390/biology10020138)
Supplement: Supplementary file 1 [file biology-10-00138-s001.pdf]

Supplementary Material

Supplementary Table S1. Fungal genus distribution.

Genus	Oceanic island: Seodo, Dokdo Islands		Coastline: Sadong, Ulleungdo Island		Inland: Taeha, Ulleungdo Island	
	**RP**	**RS**	**RP**	**RS**	**RP**	**RS**
Absidia	-	0.008	-	-	-	0.005
Acremonium	0.030	0.135	-	0.002	-	-
Agaricus	-	-	-	-	-	0.018
Agrocybe	0.161	0.120	-	-	-	-
Alatospora	-	-	-	-	-	0.029
Alternaria	0.005	2.002	-	-	-	-
Amphinema	-	-	-	0.006	-	-
Archaeorhizomyces	-	-	-	-	-	0.043
Arthrinium	0.004	0.038	-	-	-	-
Arthrobotrys	-	-	-	0.027	-	-
Arthropsis	-	-	0.226	0.251	0.001	0.004
Articulospora	0.104	0.178	-	-	-	-
Ascochyta	0.001	-	-	-	-	-
Aspergillus	-	-	0.061	0.001	-	-
Astraeus	-	-	-	-	-	0.014
Aureobasidium	0.037	0.001	0.442	0.005	-	0.008
Auxarthron	0016	-	-	-	-	-
Beauveria	0.015	0.063	-	0.003	-	0.032
Bensingtonia	-	-	-	0.001	-	-
Bullera	0.056	0.029	-	-	-	-
Capnodium	-	-	-	0.012	-	-
Capronia	0.012	0.031	-	0.002	-	0.012
Chaetomium	0.033	0.286	0.001	0.049	-	0.158
Chaetosphaeria	-	-	-	-	-	0.154
Chalara	-	0.001	0.508	0.671	1.405	0.141
Chalciporus	-	-	-	-	-	0.005
Chaunopycnis	0.020	0.014	-	-	-	-
Chloridium	-	-	0.125	0.384	-	0.086
Chrysosporium	-	0.005	0.010	0.017	-	-
Circinaria	-	-	-	-	-	0.005
Cladonia	-	-	-	-	-	0.065
Cladophialophora	0.005	0.010	-	-	0.011	0.015
Claroideoglomus	-	-	-	0.002	-	-
Clathrus	-	-	-	0.001	-	0.003
Clavaria	-	-	-	-	-	0.024
Clonostachys	-	-	-	-	-	0.003
Cochliobolus	0.059	0.070	-	-	-	-
Coniochaeta	-	-	-	0.002	-	-
Conlarium	-	0.031	-	-	-	-
Coprinellus	-	-	-	-	-	0.007

Genus	Oceanic island: Seodo, Dokdo Islands		Coastline: Sadong, Ulleungdo Island		Inland: Taeha, Ulleungdo Island	
	RP	RS	RP	RS	RP	RS
Coryneopsis	-	0.002	-	-	-	-
Crepidotus	-	-	-	-	-	0.044
Cryptococcus	1.309	0.574	-	0.024	2.213	0.008
Cryptosporiopsis	-	0.005	-	-	-	-
Cryptostroma	-	-	-	-	-	0.012
Cunninghamella	0.003	0.057	-	0.002	-	-
Cylindrocarpon	-	-	-	0.008	-	-
Cyphellophora	-	0.003	-	-	-	0.003
Cystolepiota	-	-	-	0.014	-	0.013
Derxomyces	-	-	-	-	-	0.008
Devriesia	0.061	0.018	-	-	-	0.042
Diaporthe	-	-	0.723	0.145	-	0.007
Diatrype	-	-	-	0.004	-	0.011
Dictyochaeta	-	-	-	-	-	0.017
Dioszegia	0.216	0.005	-	-	-	-
Diploicia	-	0.003	-	-	-	-
Disciotis	-	-	-	-	-	0.002
Discosia	-	0.004	-	-	-	0.020
Doratomyces	-	0.006	-	0.028	-	-
Drechslerella	-	-	-	0.024	-	-
Elaphocordyceps	-	0.003	-	-	-	-
Elsinoë	-	-	-	-	-	0.005
Entoloma	-	-	0.069	-	-	-
Eucasphaeria	-	-	-	0.012	-	-
Evlachovaea	-	-	-	-	-	0.008
Exidia	0.013	-	-	-	-	-
Exobasidium	-	-	-	0.004	-	0.010
Exophiala	1.641	0.704	-	-	-	0.297
Flammulina	-	-	-	-	-	0.002
Fusarium	1.225	1.154	0.541	0.096	0.001	0.049
Fusicladium	-	-	-	0.002	-	-
Ganoderma	-	-	0.189	0.045	-	
Geminibasidium	-	-	-	0.008	-	0.001
Geoglossum	-	-	-	0.000	-	0.006
Geomyces	0.001	-	-	-	-	-
Geosmithia	-	-	-	0.018	-	0.009
Geotrichum	-	-	0.001	-	-	-
Gibellulopsis	0.733	0.108	-	-	-	-
Gliocephalotrichum	-	-	-	0.003	-	0.002
Glomerella	0.019	0.026	-	0.004	-	0.086
Glomus	-	-	-	0.005	-	-
Gongronella	-	0.006	0.319	0.004	-	-
Gymnostellatospora	-	-	-	0.081	-	-

Genus	Oceanic island: Seodo, Dokdo Islands		Coastline: Sadong, Ulleungdo Island		Inland: Taeha, Ulleungdo Island	
	**RP**	**RS**	**RP**	**RS**	**RP**	**RS**
Haematonectria	0.012	0.001	-	-	-	-
Haptocillium	-	0.003	0.099	-	-	0.009
Hyalopeziza	-	-	-	-	-	0.006
Hydnobolites	-	-	-	-	-	0.007
Hyphoderma	-	-	0.001	-	24.018	-
Hyphodontia	-	-	-	0.004	-	-
Hypocrea	-	-	0.099	0.044	-	0.043
Hypomyces	-	-	-	0.007	-	-
Ilyonectria	-	-	0.001	0.153	-	-
Itersonilia	-	-	-	0.009	-	-
Kazachstania	-	-	-	-	-	0.005
Lecanicillium	-	0.002	-	-	-	-
Lecythophora	-	-	-	0.043	-	0.043
Lepiota	-	-	-	0.004	-	-
Leptodiscella	-	-	-	0.027	-	0.013
Leptosphaeria	-	-	0.226	-	-	-
Leptospora	0.013	-	-	-	-	-
Leucoagaricus	-	-	-	0.017	-	-
Leucosporidiella	0.007	0.055	-	-	-	-
Lipomyces	-	-	-	0.002	-	-
Lophodermium	-	-	-	0.030	-	0.019
Malassezia	0.002	-	0.020	0.002	0.333	0.006
Mariannaea	-	-	-	0.086	-	-
Massarina	-	0.012	-	-	-	-
Metacordyceps	-	0.011	-	-	-	-
Metarhizium	-	-	-	0.008	-	-
Monacrosporium	-	-	-	0.041	-	-
Monographella	0.108	0.026	1.241	0.497	-	-
Mortierella	7.780	13.064	3.541	1.732	21.260	23.204
Mrakiella	-	-	-	0.056	-	0.016
Mucor	0.259	0.515	-	-	-	0.007
Myceliophthora	-	0.024	-	-	-	-
Mycena	2.537	2.997	-	-	-	-
Myrothecium	-	-	0.068	0.004	-	0.052
Nectria	-	-	-	0.076	-	0.108
Neobulgaria	0.000	-	-	-	-	-
Neonectria	0.003	0.036	-	0.028	-	0.076
Neotyphodium	-	-	-	0.014	-	-
Ochroconis	-	-	-	-	-	0.041
Oidiodendron	-	-	0.134	-	0.001	0.032
Paecilomyces	-	-	0.224	0.304	-	0.076
Paraconiothyrium	0.032	0.032	0.350	0.689	-	-
Paraphoma	0.009	0.066	-	-	-	0.179
Penicillium	1.063	1.020	0.246	0.267	0.001	0.363

Genus	Oceanic island: Seodo, Dokdo Islands		Coastline: Sadong, Ulleungdo Island		Inland: Taeha, Ulleungdo Island	
	**RP**	**RS**	**RP**	**RS**	**RP**	**RS**
Pestalotiopsis	-	-	-	0.027	-	0.008
Phaeomoniella	0.007	0.010	-	-	-	0.015
Phaeosphaeria	0.070	-	-	0.007	-	0.041
Phialocephala	-	-	-	-	-	0.130
Phomopsis	0.005	0.024	-	-	-	0.012
Pithoascus	-	0.007	-	0.019	-	-
Pleurotheciella	-	-	0.345	0.003	-	0.254
Pochonia	-	0.023	0.101	0.024	-	0.032
Podospora	0.034	0.011	-	0.030	-	0.500
Pseudocercosporella	-	-	-	-	0.445	0.429
Pseudodictyosporium	-	-	-	-	-	0.011
Ramalina	-	-	-	-	-	0.011
Ramicandelaber	-	-	-	-	-	0.003
Ramichloridium	-	-	-	-	-	0.005
Rhexocercosporidium	-	0.008	-	-	-	-
Rhinocladiella	-	-	-	-	-	0.008
Rhizophlyctis	-	-	-	0.013	-	-
Rhizophydium	-	-	-	0.006	-	0.072
Rhodotorula	0.022	0.007	0.118	0.021	-	0.015
Roridomyces	-	-	0.432	0.014	-	-
Sagenomella	-	-	-	0.006	-	-
Sarea	-	-	-	0.008	-	0.014
Scleroderma	-	-	-	-	-	0.256
Scleromitrula	-	-	-	-	-	0.003
Sclerotinia	-	-	-	-	-	0.017
Seimatosporium	-	-	-	-	-	0.001
Setosphaeria	0.112	0.046	-	-	-	-
Simplicillium	-	-	0.003	-	-	-
Sphacelotheca	-	0.033	-	-	-	-
Sporobolomyces	0.009	-	-	0.002	-	-
Sporothrix	-	0.008	-	-	-	-
Stachybotrys	-	-	-	0.041	-	0.051
Stagonospora	5.503	0.457	-	-	-	-
Stemphylium	0.398	-	0.704	-	-	-
Stereum	0.008	-	-	-	-	-
Stilbella	-	-	-	-	-	0.011
Talaromyces	0.034	0.031	-	0.023	-	-
Teratosphaeria	0.045	0.023	-	-	-	-
Tetracladium	0.000	0.063	-	0.108	0.451	0.307
Thysanophora	-	-	-	-	-	0.012
Tolypocladium	0.015	0.018	-	-	-	-
Trechispora	-	-	-	0.001	-	-
Tremellodendropsis	-	-	-	-	-	0.008

Genus	Oceanic island: Seodo, Dokdo Islands		Coastline: Sadong, Ulleungdo Island		Inland: Taeha, Ulleungdo Island	
	**RP**	**RS**	**RP**	**RS**	**RP**	**RS**
Trichoderma	0.778	0.104	0.386	0.320	2.205	0.155
Trichosporon	-	0.383	-	-	-	0.751
Tricladium	-	0.021	-	-	-	-
Tuber	-	-	-	-	0.725	0.162
Tubeufia	-	-	-	-	-	0.004
Tyromyces	0.014	0.007	-	0.002	-	0.002
Udeniomyces	0.003	0.001	-	0.014	-	-
Umbelopsis	1.906	2.158	0.001	-	1.174	0.124
Ustilago	0.007	0.003	-	-	-	-
Veronaeopsis	-	-	-	-	-	0.032
Verrucaria	-	-	-	-	-	0.050
Volutella	0.014	0.002	-	0.003	-	0.013
Waitea	-	-	-	0.003	-	-
Wardomyces	-	-	-	-	-	0.003
Xenopolyscytalum	-	-	-	-	-	0.008

RP: rhizoplane, RS: rhizosphere

Supplementary Table S2. Soil analysis results.

**Soil**	**Particle size distribution**			**Soil texture class**	**pH**	**Organic material (%)**	**Total nitrogen (%)**	**Sodium chloride (%)**
	**Sand (%)**	**Silt (%)**	**Clay (%)**					
Oceanic island: Seodo, Dokdo Islands	57.4	39.4	3.2	Sandy soil	5.0	7.6	0.52	0.012
Coastline: Sadong, Ulleungdo Island	32.0	64.8	3.2	Silt	7.4	13.4	0.64	0.005
Inland: Taeha, Ulleungdo Island	49.8	46.7	3.5	Sandy soil	6.7	4.8	0.26	0.003
